# Mesoporous hollow carbon spheres for lithium–sulfur batteries: distribution of sulfur and electrochemical performance

**DOI:** 10.3762/bjnano.7.114

**Published:** 2016-08-30

**Authors:** Anika C Juhl, Artur Schneider, Boris Ufer, Torsten Brezesinski, Jürgen Janek, Michael Fröba

**Affiliations:** 1 Institute of Inorganic and Applied Chemistry, University of Hamburg, Martin-Luther-King-Platz 6, 20146 Hamburg, Germany; 2 Battery and Electrochemistry Laboratory, Institute of Nanotechnology, Karlsruhe Institute of Technology, Hermann-von-Helmholtz-Platz 1, 76344 Eggenstein-Leopoldshafen, Germany; 3 Institute of Physical Chemistry, Justus-Liebig-University Giessen, Heinrich-Buff-Ring 17, 35392 Giessen, Germany

**Keywords:** carbon/sulfur composites, cycling stability, distribution of sulfur in pores, hollow carbon spheres, lithium–sulfur batteries

## Abstract

Hollow carbon spheres (HCS) with a nanoporous shell are promising for the use in lithium–sulfur batteries because of the large internal void offering space for sulfur and polysulfide storage and confinement. However, there is an ongoing discussion whether the cavity is accessible for sulfur. Yet no valid proof of cavity filling has been presented, mostly due to application of unsuitable high-vacuum methods for the analysis of sulfur distribution. Here we describe the distribution of sulfur in hollow carbon spheres by powder X-ray diffraction and Raman spectroscopy along with results from scanning electron microscopy and nitrogen physisorption. The results of these methods lead to the conclusion that the cavity is not accessible for sulfur infiltration. Nevertheless, HCS/sulfur composite cathodes with areal sulfur loadings of 2.0 mg·cm^−2^ were investigated electrochemically, showing stable cycling performance with specific capacities of about 500 mAh·g^−1^ based on the mass of sulfur over 500 cycles.

## Introduction

In the past 20 years, rechargeable lithium–ion batteries have proven to be superior energy storage devices and have been subject of intensive research [1–3]. However, being limited by a theoretical specific capacity of the active materials of approximately 300 mAh·g^−1^, their storage capacity is not sufficient to serve as the primary energy source of domains such as long-range automotive transport [4–5]. Due to the high theoretical specific capacity (1675 mAh·g^−1^) and specific energy (2600 Wh·kg^−1^) of sulfur the lithium–sulfur (Li–S) battery is a promising candidate to overcome this limitation and, thus, replace the Li–ion system [4,6]. Besides, sulfur offers the advantages of being naturally abundant, non-toxic and of low cost.

Nevertheless, the Li–S cell is facing several problems that have to be settled for industrial application. One is the insulating nature of sulfur and its discharge product lithium sulfide (Li_2_S), which leads to a low utilization of active material [7–9]. Another problem is the solubility of the lithium polysulfides (Li_2_S*_x_*, 3 ≤ *x* ≤ 8) formed as intermediate products during charge and discharge in the commonly used organic electrolytes. The dissolved polysulfides shuttle between the cathode and anode and cause the deposition of insoluble Li_2_S_2_ and Li_2_S on both upon further reduction at the end of discharge. In consequence, the cell suffers from low Coulombic efficiency and short cycle life [8,10–11]. The third drawback is a volume expansion of about 80% during discharge, resulting from the lower density and thus higher molar volume of lithium sulfide (28.0 cm^3^·mol^−1^ compared to 15.5 cm^3^·mol^−1^ for sulfur) [12]. This can lead to the loss of electrical contact of Li_2_S with the conducting additive or the current collector [9].

Cathode materials composed of porous carbon and sulfur show promising results with regard to overcoming these problems. Thus, a lot of research has been carried out on nanostructured carbon hosts for sulfur storage including carbon fibers [13–14], carbon nanotubes [15–16], graphene/graphene oxide [17–19] as well as micro-/mesoporous carbons [20–22]. Among the porous carbons, especially hollow carbon spheres (HCS) have attracted significant attention because sulfur and the resulting polysulfides can be confined in the shell while the large cavity offers room for sulfur storage and volume expansion during discharge [23–31].

However, there is an ongoing discussion on the location of sulfur in the hollow spheres. It remains unclear at present, whether the cavity and the micro- or mesopores of the shell are both accessible for sulfur infiltration. The analysis of sulfur distribution in the literature is usually conducted by energy dispersive X-ray spectroscopy (EDX) measurements using either a transmission electron microscope (TEM) or a scanning electron microscope (SEM). Although revealing similarities in the main characteristics, the conclusions being drawn are rather contradictory [24–31]. A problem may arise from the high spatial mobility of sulfur species under vacuum conditions. Raiß et al. examined the behavior of sulfur in the presence of carbon in high vacuum and found that sulfur is redistributing rapidly. They concluded that for this reason, the analysis of carbon/sulfur composites by means of vacuum-based methods can be misleading [32].

In this work, we present the analysis of sulfur distribution in hollow carbon spheres with a mesoporous shell by combining the results from non-vacuum methods, namely X-ray diffraction (XRD) and Raman spectroscopy, with those from vacuum-based ones (SEM and nitrogen physisorption). Moreover, we examined the influence of the pressure during melt impregnation on the distribution of sulfur and compared the resulting loading and distribution with composites obtained by impregnation from a solution of sulfur in carbon disulfide. Finally, the electrochemical performance of HCS/sulfur composite cathodes with a sulfur areal loading of 2.0 mg·cm^−2^ was investigated.

## Results and Discussion

### Silica template and hollow carbon spheres

**Figure 1:**
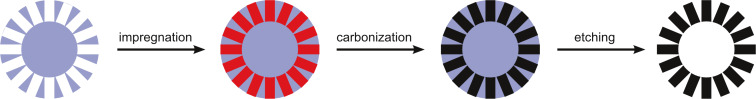
Synthesis of the hollow carbon spheres via impregnation of silica spheres with solid core and mesoporous shell, followed by carbonization and etching of silica.

Hollow carbon spheres with a mesoporous shell were obtained by impregnation of silica spheres with a core–shell structure with phenol and formaldehyde (first step in Figure 1). Carbonization under inert atmosphere and etching of the template yielded the hollow spheres (second and third step in Figure 1).

The employed silica spheres with a solid core and mesoporous shell (SCMS silica) were synthesized in two steps by modified literature methods [33–34]. Solid silica spheres were synthesized by the Stöber method [35]. In the second step a mesoporous shell was grown on the spheres by employing tetraethyl orthosilicate in presence of cetyltrimethylammonium bromide (CTAB) as a structure-directing agent. Combustion of CTAB in air generated the core–shell silica spheres. The diameter of the solid core was determined to be 380 nm by dynamic light scattering, while the diameter of the core–shell particles was about 515 nm. From SEM images (Figure S1 in Supporting Information File 1) a diameter of about 490 nm was determined for the core–shell spheres. Further characterization of the SCMS silica can be found in Supporting Information File 1 (Figure S2).

**Figure 2:**
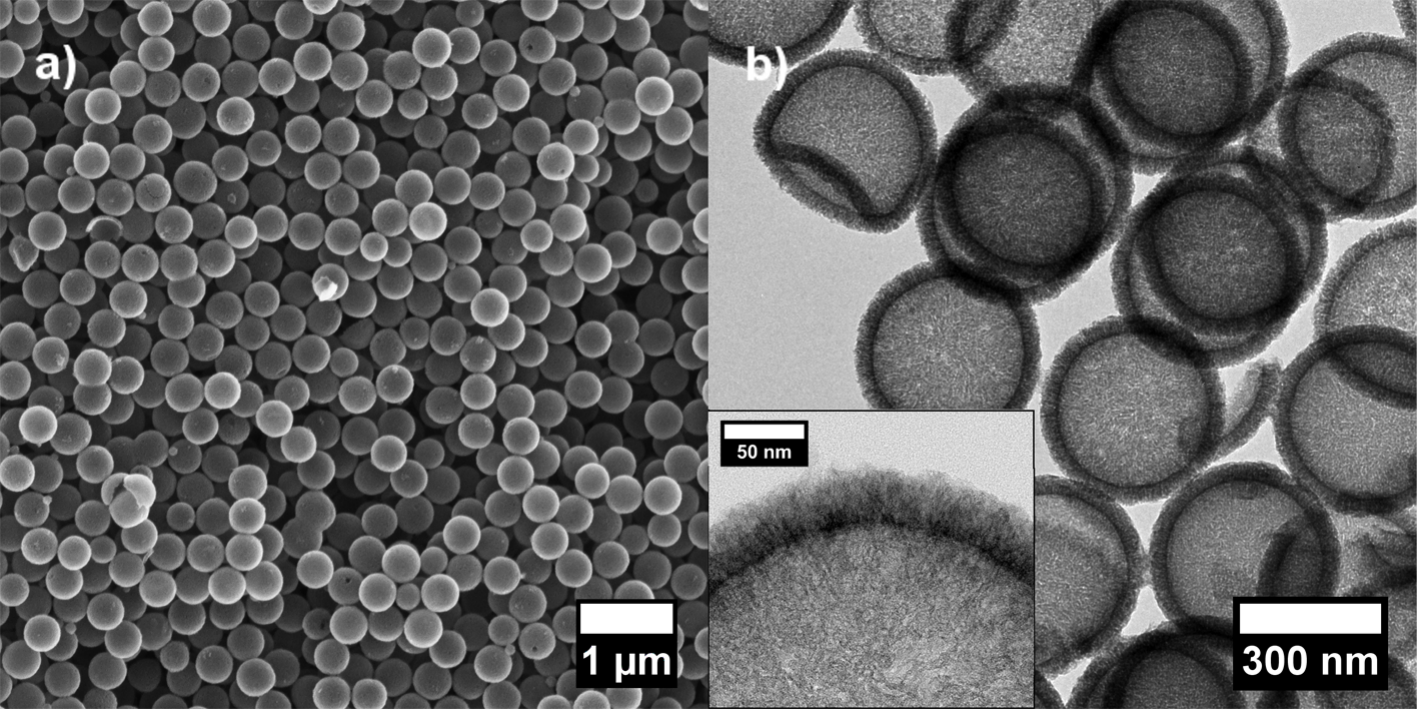
a) SEM and b) TEM images of mesoporous hollow carbon spheres. Inset: larger magnification of the shell of a hollow sphere.

**Figure 3:**
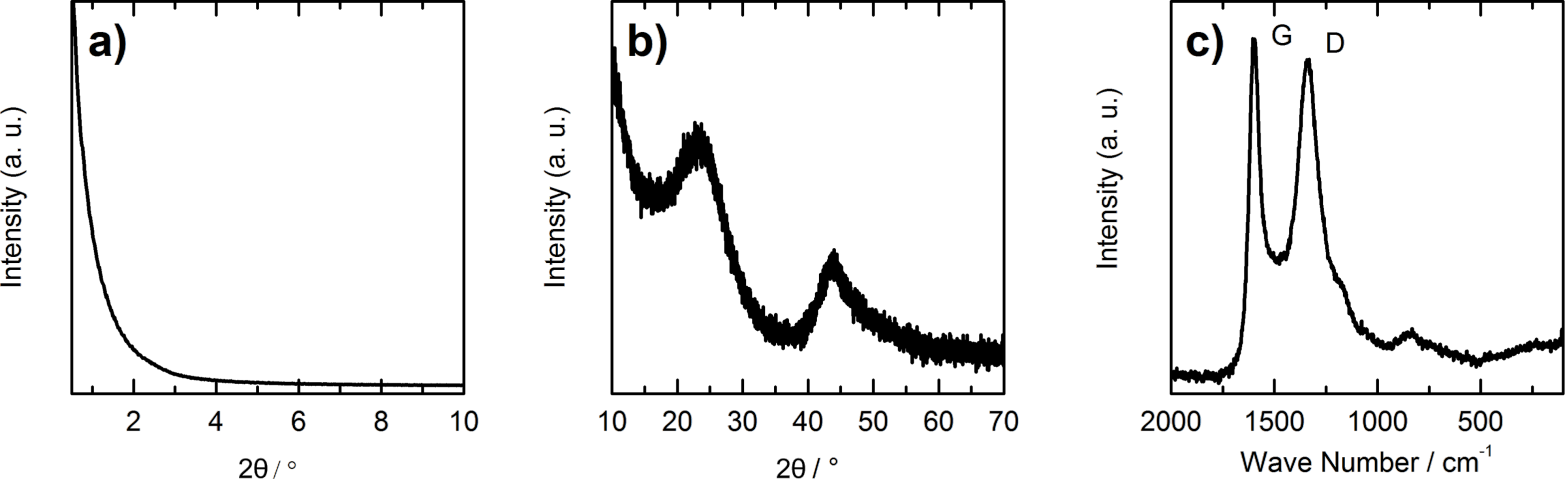
a) Small-angle and b) wide-angle XRD patterns as well as c) Raman spectrum of hollow carbon spheres.

SEM and TEM images of HCS synthesized from SCMS silica (Figure 2) show that the particle size is uniform with an outer diameter of approximately 400 nm, an inner diameter of about 300 nm and a shell thickness of roughly 40 to 50 nm. Moreover, the shell of the HCS has an appearance typical of a material composed of disordered mesopores. The disordered structure is supported by the small-angle X-ray diffraction pattern of the hollow spheres (Figure 3a), which does not show any reflections between 2θ = 0.5° and 2θ = 10°.

The wide-angle X-ray diffraction pattern (Figure 3b) shows two broad reflections that result from the interlayer and intralayer scattering of graphene sheets. From the fact that they are broad and little pronounced it can be concluded that the degree of graphitization is low and the carbon is mainly amorphous [36–37]. This is confirmed by the Raman spectrum (Figure 3c), which shows two bands centered at 1597 cm^−1^ (G band) and 1340 cm^−1^ (D band) typical of carbon with small graphitic domains. The G band results from the in-plane stretching of sp^2^-bonded (graphitic) carbon atoms, while the D band is induced by defects and disorder in the carbon structure [38].

**Figure 4:**
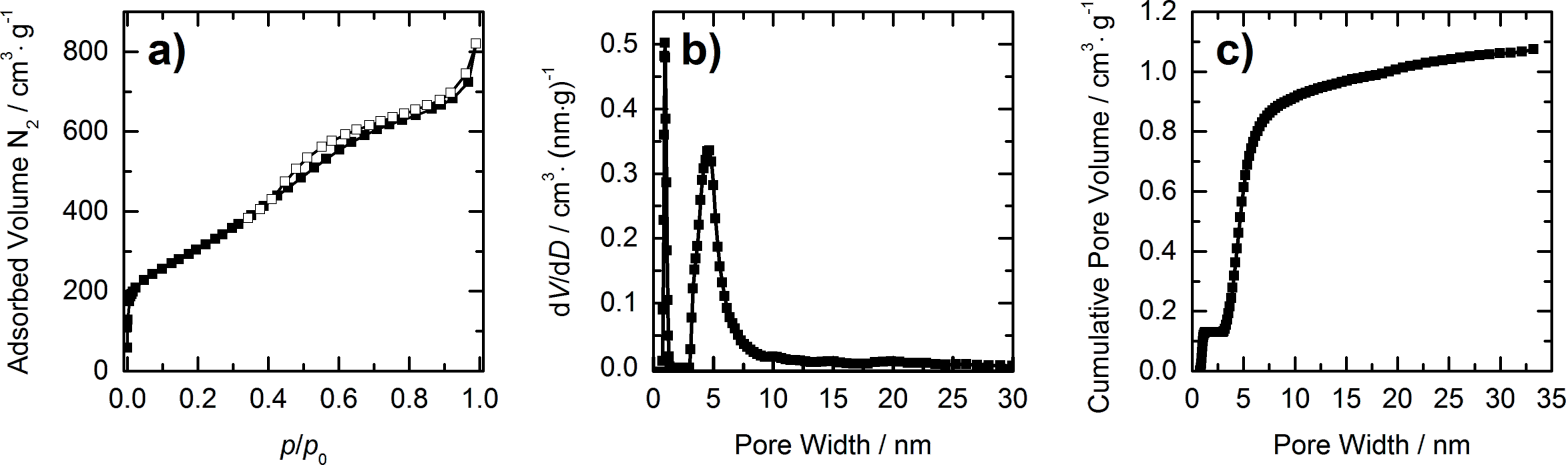
a) Nitrogen physisorption isotherm (measured at 77 K), b) pore size distribution and c) cumulative pore volume of hollow carbon spheres. Pore size distribution and cumulative pore volume were obtained from the isotherm by QSDFT analysis.

From the nitrogen physisorption isotherm (Figure 4a) of the HCS a Brunauer–Emmet–Teller (BET) surface of 1123 m^2^·g^−1^ can be determined. The pore size distribution (Figure 4b) was calculated by a quenched solid density functional theory (QSDFT) model from the adsorption branch and shows that the mesopore diameter of the spheres is approximately 5 nm. The adsorbed volume of nitrogen gas is not reaching a plateau at high relative pressures but is increasing steeply at a pressure of *p*/*p*_0_ higher than 0.9. This is because of the large inner cavity of the hollow spheres, the volume of which cannot be determined by nitrogen physisorption. And this in turn makes it impossible to determine the total pore volume of the HCS [39]. To assess the pore volume of the shell, however, the cumulative pore volume (Figure 4c) of pores in the meso-range can be used as an estimate. As the shell thickness is about 40 nm, it can be assumed that the pore volume of the shell originates from pores smaller than that. To be sure not to take into account too much of the cavity volume, the pore volume of the shell was estimated from the cumulative pore volume of pores up to 30 nm, which adds up to 1.06 cm^3^·g^−1^. The micropores (pores smaller than 2 nm) contribute 0.13 cm^3^·g^−1^ to this volume.

The microporosity of carbonaceous materials can be determined more appropriately by carbon dioxide physisorption. From carbon dioxide physisorption measurements (see Figure S3 in Supporting Information File 1 for the isotherm, pore size distribution and cumulative pore volume) it can be concluded that the HCS contain a considerable amount of pores smaller than 1.5 nm. The cumulative pore volume of these small pores is as high as 0.26 cm^3^·g^−1^, which is significantly higher than the 0.13 cm^3^·g^−1^ determined by nitrogen physisorption for pores smaller than 2 nm.

### Carbon/sulfur composites

To get an impression of how much sulfur can be loaded into the pores and the cavities of the hollow carbon spheres, we calculated which sulfur loadings can be reached by either filling only the pores of the shell of the HCS or by filling the pores of the shell and the cavity.

The maximum mass of sulfur *m*_sulfur_ that can be incorporated into the shell by melt impregnation can be calculated by multiplication of the pore volume *V*_pores_ of the shell (1.06 cm^3^ for 1 g HCS) with the density of liquid sulfur (ρ_sulfur_ = 1.819 g·cm^−3^) [12].

[1]
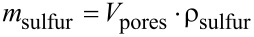


This means, that by filling just the shell of HCS, 1.93 g sulfur can be loaded into 1 g of HCS, corresponding to a sulfur loading *w*_sulfur_ of 65 wt %. The latter value was calculated by using Equation 2, where *m*_HCS_ is the mass of the hollow carbon spheres.

[2]
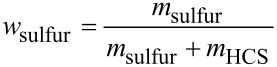


The maximum sulfur loading for the case that both the cavity and the shell of HCS are filled by sulfur can also be calculated when considering the pore volume of the shell *V*_pores_ and the volume of the cavities by taking into account the geometry of a sphere. The derivation of the resulting Equation 3 can be found in Supporting Information File 1. The constant *C* is given by Equation 4.

[3]
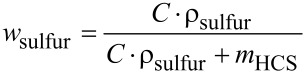


[4]
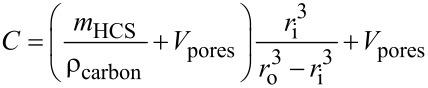


In Equation 4, ρ_carbon_ represents the density of carbon without pores, *r*_i_ is the radius of the cavity (the inner radius of the hollow sphere) and *r*_o_ is the outer radius of the sphere.

Because the maximum sulfur loading by melt impregnation is calculated, the density of liquid sulfur is used again. The density of the carbon is unknown, but as the HCS are obtained by carbonization at 900 °C and the XRD pattern indicates mainly amorphous carbon, the density of amorphous carbon (ρ_amorphousC_ = 1.8 g·cm^−3^) [12] is assumed. This way a sulfur loading of 81 wt % is obtained. For comparison, the possible sulfur loadings were also determined using the densities of graphite (ρ_graphite_ = 2.30 g·cm^−3^) [12] and charcoal (ρ_charcoal_ = 0.56 g·cm^−3^) [12]. These calculations led to values of 80 wt % and 86 wt %, respectively. Consequently, it can be concluded that a sulfur loading between 80 wt % and 86 wt % can be reached by filling both the shell and the cavity of HCS with sulfur.

To monitor the filling of the hollow spheres, they were loaded with sulfur in mass ratios of carbon to sulfur ranging from 70:30 to 30:70 by a melt impregnation method at 155 °C. For comparison, samples with sulfur loadings of 60 wt % and 70 wt % were also prepared in vacuum and under increased pressure. Moreover, impregnation of HCS with sulfur from a solution containing sulfur in carbon disulfide was carried out repeatedly so that sulfur loadings close to 60 and 70 wt % were obtained. The mass fractions of carbon and sulfur in the composites were determined by thermogravimetric measurements (see Figure S5 in Supporting Information File 1) and the corresponding values are given in Table 1. Samples are denoted as HCS-*x*-*method*, where *x* indicates the exact amount of sulfur and *method* specifies the impregnation method. The term “melt” stands for melt impregnation at ambient pressure, “vac” for melt impregnation in vacuum, “press” indicates impregnation under increased pressure and “sol” stands for the impregnation from solution. It is worth noting that even the composite with the highest fraction of sulfur (HCS-76-sol) contains less sulfur than can theoretically be filled into the pore volume of cavity and shell, which would be at least 80 wt % (see calculations above).

**Table 1:** Water, sulfur and carbon content of HCS, HCS/sulfur composites and elemental sulfur determined by thermogravimetric measurements as well as the residual mass after thermogravimetry.^a^

sample	water content / %	sulfur content / %	carbon content / %	residual mass / %

HCS	2.3	—	96.0	2.0
elemental sulfur	—	99.5	—	0.6
HCS-29-melt	1.0	29.2	68.3	1.9
HCS-38-melt	0.6	37.9	60.6	0.8
HCS-49-melt	—	48.6	50.5	0.5
HCS-59-melt	0.2	59.1	39.2	1.5
HCS-68-melt	—	68.3	30.1	1.6
HCS-58-vac	—	58.0	41.2	1.0
HCS-67-vac	—	66.5	31.7	2.3
HCS-59-press	—	58.8	39.5	1.2
HCS-67-press	—	67.2	31.2	2.2
HCS-53-sol^b^	—	53.0	45.4	1.2
HCS-76-sol^b^	—	76.0	23.0	1.7

^a^Values that add up to more than 100% are due to the measuring inaccuracy of thermogravimetry. ^b^Mass ratios obtained by impregnation from solution differ from those obtained by the other methods because carbon disulfide evaporated fast, thus changing the concentration of the solution.

#### X-ray diffraction

**Figure 5:**
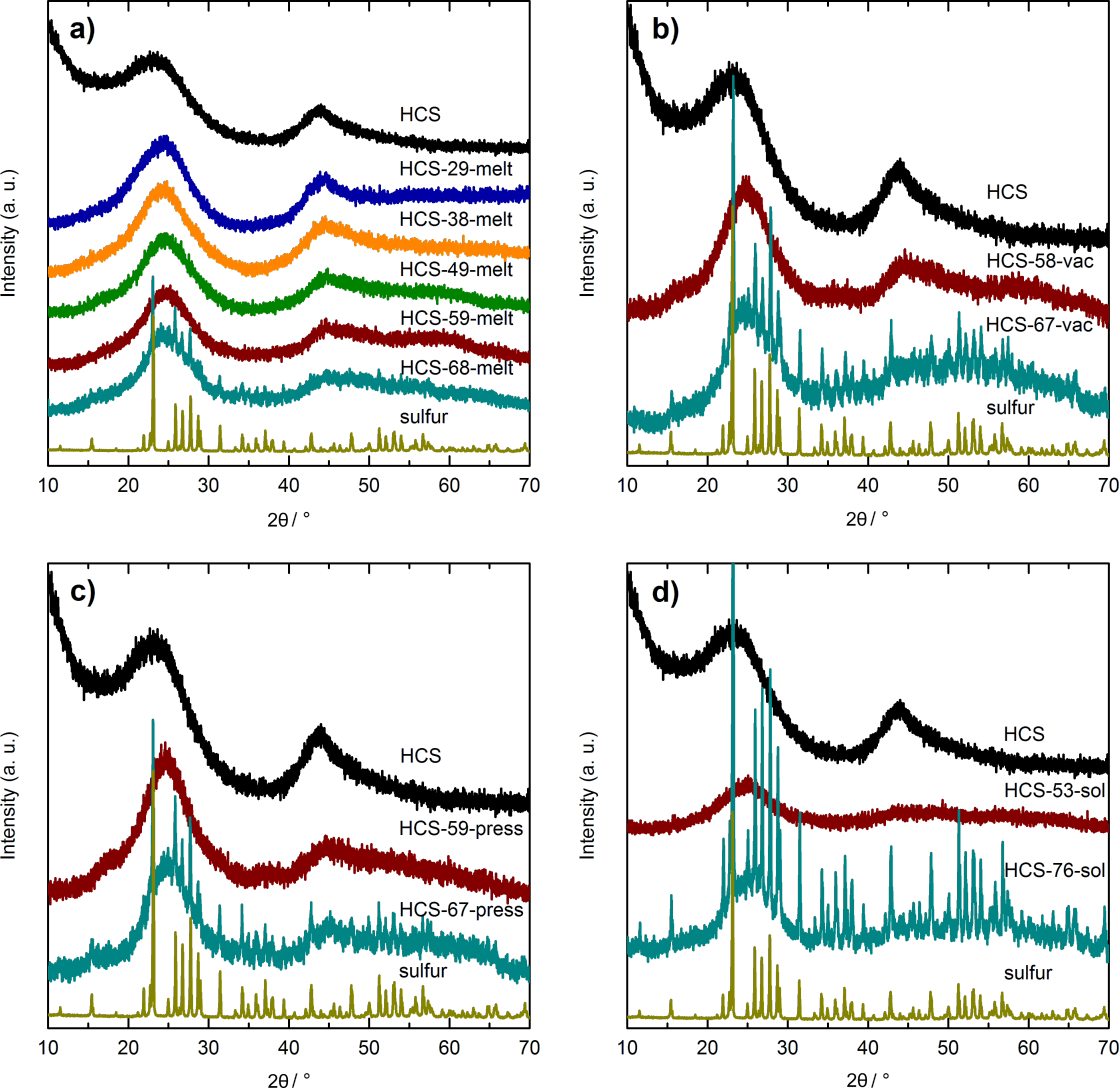
Powder X-ray diffraction patterns of HCS loaded with different amounts of sulfur by a) melt impregnation at ambient pressure, b) melt impregnation in vacuum, c) melt impregnation under increased pressure and d) impregnation from a solution of sulfur in carbon disulfide.

The HCS/sulfur composites were investigated by powder X-ray diffraction (pXRD). The wide-angle XRD patterns of the samples impregnated at ambient pressure (Figure 5a) show that up to a sulfur content of 60 wt % there are only the broad reflections of amorphous carbon visible. In the literature this behavior is attributed to sulfur being dispersed in mesopores, thereby losing its crystallinity [22,40]. For a sulfur content of 70 wt % however, reflections of crystalline sulfur can be seen. For the samples impregnated with sulfur in vacuum (Figure 5b), under increased pressure (Figure 5c) and from solution (Figure 5d), the XRD patterns look similar: For a sulfur content of less than 60 wt % no reflections are visible, while diffraction peaks of crystalline sulfur can be observed when the sulfur content is approximately 70 wt %.

There are two possible explanations for the existence of crystalline sulfur in the composites. The first is that sulfur fills the cavities of the HCS leading to larger crystallites than in the mesopores of the shell. The second explanation is that sulfur accumulates on the outside of the hollow spheres, thus showing bulk-like behavior. Given that no broadening of the sulfur reflections due to nano-sized crystallites can be observed, we conclude that the crystalline sulfur is only present on the outside of HCS.

#### Raman spectroscopy

**Figure 6:**
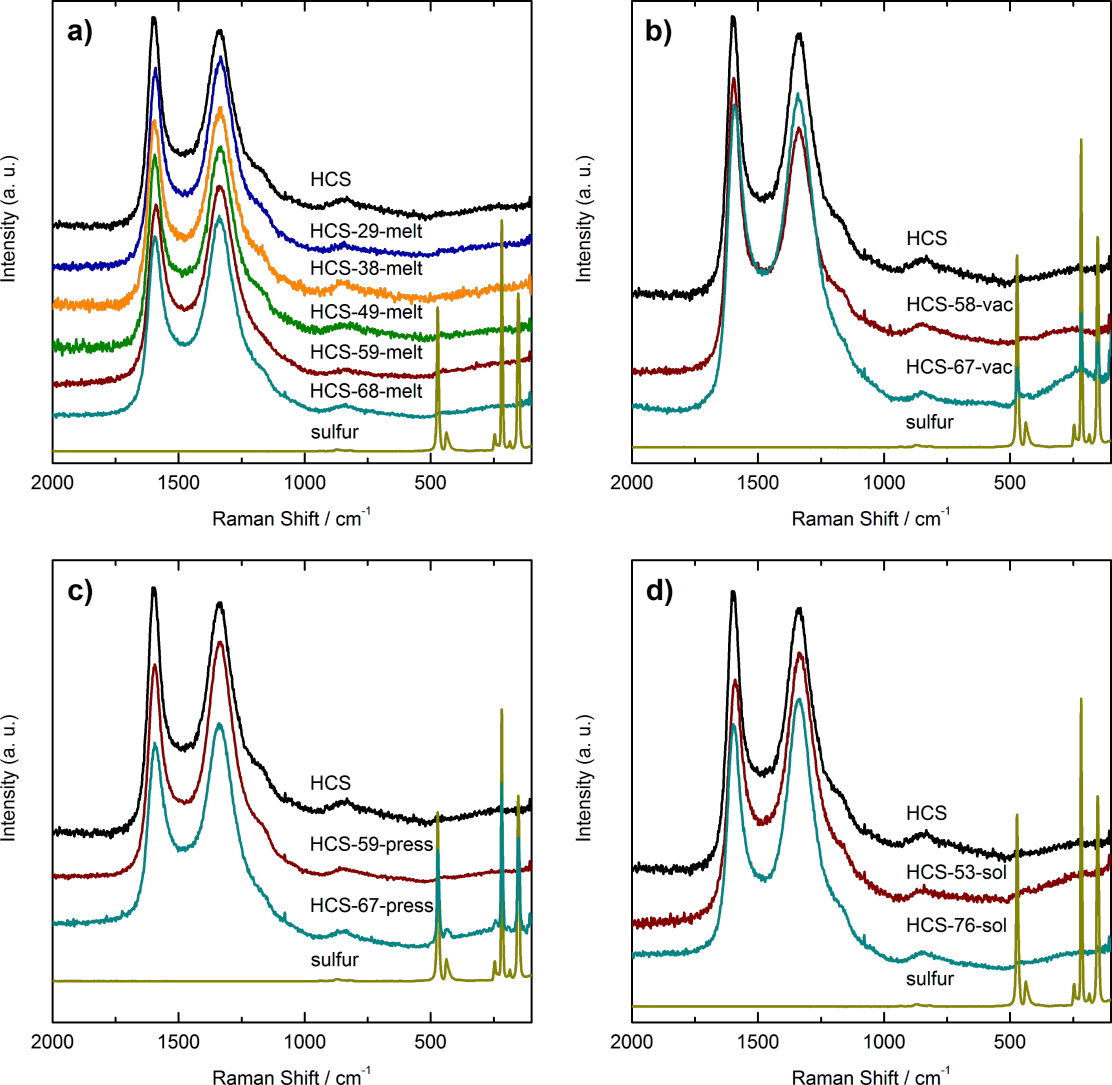
Raman spectra of HCS loaded with different amounts of sulfur by a) melt impregnation at ambient pressure, b) melt impregnation in vacuum, c) melt impregnation under increased pressure and d) impregnation from a solution of sulfur in carbon disulfide.

Raman spectroscopy (Figure 6) shows results similar to those obtained by pXRD measurements. For carbon/sulfur composites with sulfur contents below 60 wt %, the characteristic bands resulting from the vibrations of S_8_ cannot be observed, while they are visible at least in some of the composites containing more than 60 wt % sulfur. This behavior has also been reported in the literature [41–43] but could not yet be explained satisfactorily. However, as these observations are in good agreement with the results from pXRD, it can be assumed, that the lack of Raman bands might also be due to confinement effects. Thus, the appearance of bands at high sulfur loadings could also be explained either by sulfur filling the cavities of HCS or by sulfur accumulating on the outside of the spheres.

#### SEM and EDX

**Figure 7:**
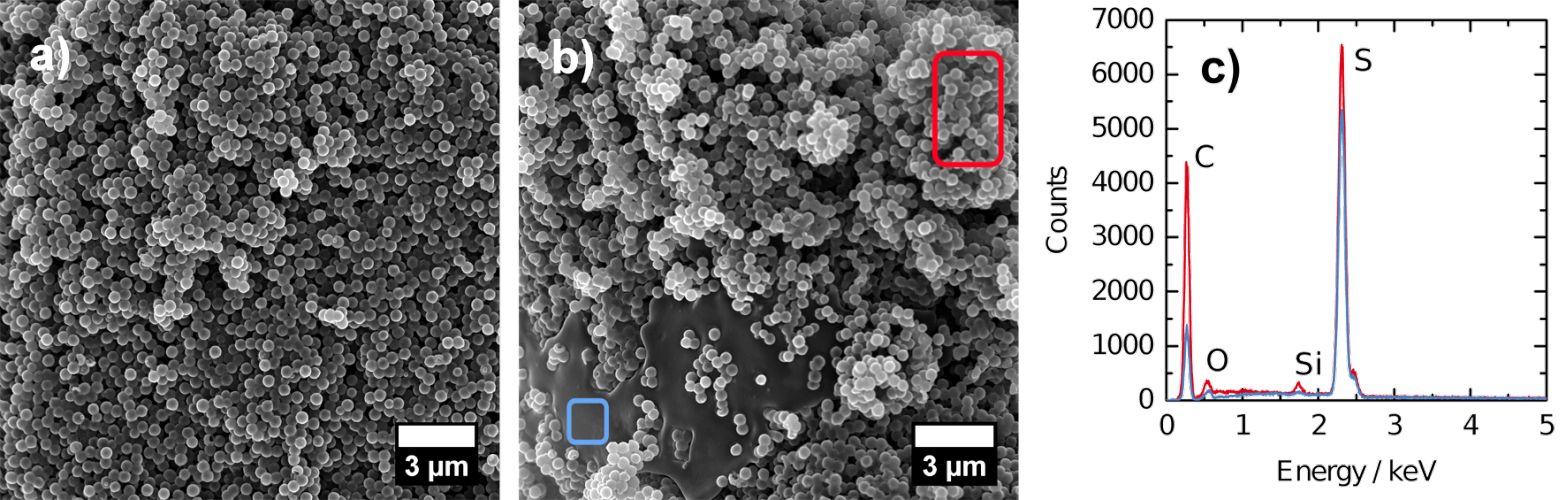
SEM images of a) HCS-58-vac, b) HCS-67-vac, and c) EDX spectra of HCS-67-vac (measured areas are marked red and blue, respectively).

SEM images of HCS-58-vac and HCS-67-vac (Figure 7a,b) which can be seen as representative examples of the composites studied here (see Figure S6 and Figure S7 in Supporting Information File 1 for the others), show that the sample morphology is significantly different depending on the sulfur content. SEM images of samples containing less than 60 wt % sulfur only show carbon spheres regardless of the impregnation method. SEM images of the composites containing 67 wt % sulfur or more indicate the presence of a second phase. This phase is darker than the spheres and is extended over large areas. Exemplary, EDX measurements of the hollow spheres and the second phase in HCS-67-vac are shown in Figure 7c; the measured areas and corresponding EDX spectra are marked in red and blue. When comparing the EDX peaks of sulfur and carbon in both areas, it can be clearly seen that there is a larger amount of sulfur present in the darker areas than in the sulfur-loaded HCS. Thus, it can be assumed that the second phase consists of molten and recrystallized sulfur. The amount of carbon that is still measureable in this area is due to the deposition of an additional carbon layer onto the samples prior to EDX analysis. EDX spectra for HCS-68-melt, HCS-67-press and HCS-76-sol can be found in Supporting Information File 1 (Figure S7). As these large accumulations of sulfur can only be found in samples with the highest sulfur contents, we assume that this result is not due to sulfur redistribution in vacuum during the SEM/EDX analysis. It also helps to explain the appearance of sulfur reflections in pXRD and characteristic sulfur bands in the Raman spectra. Keeping in mind that the shell of HCS is supposed to hold about 66 wt % sulfur while the complete hollow spheres should be able to contain as much as 80 wt % sulfur, we conclude from the results with pXRD, Raman spectroscopy and SEM/EDX that only the shell of HCS can be filled by sulfur. Using more sulfur to eventually fill the cavity leads to crystalline sulfur on the outside of HCS.

#### Nitrogen physisorption

Nitrogen physisorption measurements of the composites also confirm the inaccessibility of the HCS cavity for sulfur. Nevertheless, the results have to be handled with care because of the vacuum applied while degassing and measuring the samples. From the physisorption isotherms the pore volume of the shell of the HCS/sulfur composites can be determined in the same way as that of the pure hollow spheres (see Figure S8 in Supporting Information File 1 for the physisorption isotherms and plots of cumulative pore volumes). For comparison, a theoretical pore volume can be calculated from the pore volume of HCS and the volume of the impregnated sulfur according to Equation 5 (for the derivation see Supporting Information File 1). The density of liquid sulfur was used for the calculation of the theoretical pore volumes because both the state and structure of sulfur in nanopores are unknown.

[5]
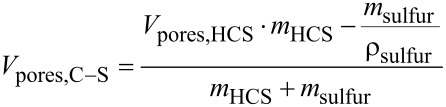


The calculation was carried out for all samples containing less than 60 wt % sulfur as the others contain a sulfur volume higher than the pore volume of the shell. Table 2 gives the pore volumes of HCS and HCS/sulfur composites determined from nitrogen physisorption and the calculated pore volumes. Interestingly, the measured pore volume is always lower than the calculated value. This might be due to the fact that some pores are blocked by sulfur and thus, are no longer accessible for nitrogen gas during the measurements. We believe that the strong capillary forces on liquids/melts in the mesopores are responsible for the pore blocking effect. Once sulfur has filled the mesopores there is no further driving force for filling of the cavities, and only the mesoporous shell is filled with sulfur.

**Table 2:** Pore volume of HCS and HCS/sulfur composites measured by nitrogen physisorption and theoretical pore volume of the composites calculated from the pore volume of HCS and the volume of the impregnated amount of sulfur.

sample	measured pore volume / cm^3^·g^−1^	theoretical pore volume / cm^3^·g^−1^

HCS	1.06	—
HCS-29-melt	0.57	0.58
HCS-38-melt	0.39	0.44
HCS-49-melt	0.23	0.27
HCS-59-melt	0.08	0.09
HCS-58-vac	0.07	0.12
HCS-59-press	0.08	0.10
HCS-53-sol	0.17	0.19

### Electrochemical characterization

**Figure 8:**
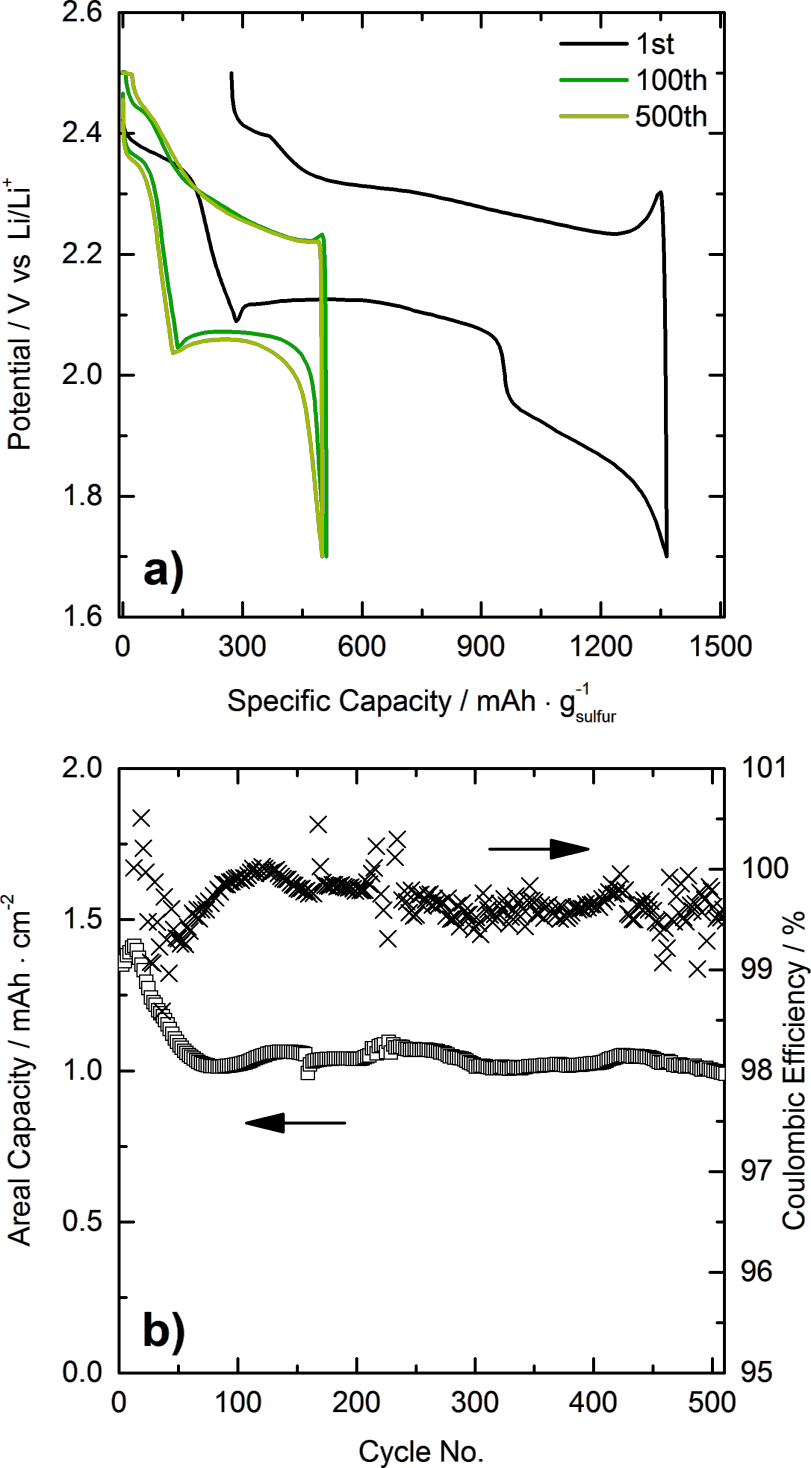
a) Voltage profiles of a Li–S cell with areal sulfur loading of 2.0 mg·cm^−2^. After the formation cycle at C/50, the rate was increased to C/5. b) Areal capacity and Coulombic efficiency versus the cycle number. The 1st cycle areal capacity was about 2.8 mAh·cm^−2^.

Since we could show that, for a HCS/sulfur ratio of approximately 40:60, the sulfur is completely incorporated in the porous carbon, electrochemical testing was performed on coin-type cells using HCS/sulfur composite containing 61 wt % sulfur as cathode material. The voltage range was 2.5–1.7 V with respect to Li/Li^+^. Representative charge–discharge curves for the first, 100th and 500th cycles of a cell with areal sulfur loading of 2.0 mg·cm^−2^ are shown in Figure 8a. This intermediate loading was chosen in order to ensure competitive areal capacities. Moreover, electrodes with higher sulfur loadings, especially with increased electrode thickness are prone to severe degradation, as the mechanical stress and the interfacial resistance are increased. Two distinct plateaus at about 2.3 V and 2.1 V are clearly visible upon discharge, corresponding to the reduction of elemental sulfur to higher-order lithium polysulfides (Li_2_S*_x_* with 6 ≤ *x* ≤ 8) and formation of lower-order lithium polysulfide species (Li_2_S*_y_* with 2 ≤ *y* ≤ 6) and Li_2_S, respectively. The plateau at 1.8 V in the first discharge cycle at C/50 rate can be ascribed to lithium nitrate decomposition at the cathode side [44]. The charge curve shows two plateaus at about 2.2 V and 2.4 V, indicating the reoxidation of lithium polysulfides to sulfur. Figure 8b presents data on the long term performance at C/5. As can be seen, the specific capacity (areal capacity) levels off at about 500 mAh·g^−1^ (1 mAh·cm^−2^) after 60 cycles, and the cell exhibits rather stable performance for 500 cycles with a fade rate of 0.06% per cycle (between the 2nd and 500th cycle at C/5). Also, the Coulombic efficiency stabilizes above 99.5%, thereby indicating good reversibility.

The measured areal capacities are comparable with those calculated from literature data [24,27,29]. Nevertheless, due to differences in cell type, electrode and electrolyte composition as well as electrolyte/sulfur ratio, a precise comparison is not possible. Even more, as necessary information for this comparison like the electrolyte/sulfur ratio and partly also the areal sulfur loading are often not given.

Overall, the data in Figure 8 demonstrate that Li–S cells based on HCS/sulfur composite show good cyclability, with moderate specific capacities at C/5 rate. Given that the results were obtained on non-optimized cathodes, this is a good starting point for future research in this direction.

## Conclusion

Hollow carbon spheres with a mesoporous shell were loaded with different amounts of sulfur by melt impregnation at ambient pressure, in vacuum and under increased pressure as well as from a solution of sulfur in carbon disulfide. By combining calculations considering the mesopore volume of the shell and the size of the cavity with results from pXRD, Raman spectroscopy and SEM, it could be concluded that the cavity of HCS is not filled by sulfur regardless of the impregnation method. This is an important result as the analysis of HCS/sulfur composites is usually carried out by EDX measurements during which sulfur can redistribute due to the vacuum applied. Although the cavity of the HCS remains empty during sulfur loading, batteries using HCS/sulfur composite with 61 wt % sulfur and with reasonably high sulfur loading of 2.0 mg·cm^−2^ showed stable electrochemical performance over 500 cycles. It seems possible that the empty cavity has a positive effect on polysulfide confinement. In summary, the HCS employed in this work are a promising system capable of storing and retaining significant amounts of sulfur, thus ensuring stable performance upon prolonged cycling.

## Experimental details

### Synthesis of hollow carbon spheres

#### Synthesis of silica template

Silica spheres with a solid core and mesoporous shell were synthesized according to a modification of literature methods in a two-step procedure [33–34]. For synthesis of the core, 536 mL of ethanol were mixed with 45 mL of aqueous ammonia (32%) and 6.8 mL of deionized water. After stirring for 30 min, 24 mL of tetraethyl orthosilicate (TEOS) were added and the mixture was stirred at room temperature for 18 h. The resulting suspension was diluted with 1200 mL of deionized water and 180 mL of a solution of cetyltrimethylammonium bromide (0.11 mol/L in a 2:1 mixture of water and ethanol) was added. The suspension was stirred at room temperature for one hour before 13.5 mL of TEOS were added dropwise. After stirring for another 18 h at room temperature, the suspension was neutralized with hydrochloric acid (32%) to precipitate the core–shell particles. The precipitate was centrifuged and dried at 60 °C before removing the surfactant by calcination at 550 °C for 6 h in air.

#### Synthesis of hollow carbon spheres

The synthesis of hollow carbon spheres was carried out by a combination and modification of literature methods [45–46]. Phenol (3.52 g) was melted at 45 °C and 615 μL of a 20 wt % sodium hydroxide aqueous solution were added to the liquid phenol. The solution was stirred for 15 min (with a KPG stirrer). After adding 5.6 mL of formalin (37 wt % formaldehyde) and further stirring for 5 min, 9.59 g of ground silica template were added. The mixture was heated to 75 °C and stirred for 1.5 h. The polymer/silica composite was dried in vacuum and polymerized in an oven at 100 °C for 24 h. The particles were washed with water and dried at 60 °C. Carbonization was carried out in a tubular furnace under argon atmosphere in two steps: The sample was heated to 350 °C for 5 h (heating rate: 1 °C/min) and 900 °C for 2 h (heating rate: 5 °C/min). The silica template was removed by washing with hydrofluoric acid (10%). The samples were washed with deionized water and ethanol and dried at 100 °C. The removal of the silica template was assured by thermal combustion of the carbon in air. The template was considered removed when the residual mass was less than 2 wt %.

### Preparation of carbon/sulfur composites

Carbon/sulfur composites were prepared in four different ways.

1. Melt impregnation: HCS and sulfur were ground together in distinct weight ratios, sealed in a flask and heated to 155 °C for 12 h.
2. Melt impregnation in vacuum: HCS and sulfur were ground together in distinct weight ratios, sealed in a flask, evacuated to a pressure of 1.1∙10^−4^ bar and heated to 125 °C for 12 h at this pressure.
3. Melt impregnation under pressure: HCS and sulfur were ground together in distinct weight ratios and placed in a Teflon lined steel autoclave filled to 90% capacity with water. The autoclave was sealed and the sample was heated to 155 °C for 12 h. This temperature and degree of filling with water creates a pressure of approximately 7 bar (measured by heating water in a microwave and monitoring the resulting pressure).
4. Impregnation from solution: HCS were ground for several minutes with a 0.62 M solution of sulfur in carbon disulfide. The volume of solution was chosen in accordance to the absolute pore volume of the applied amount of carbon. After drying the composite at room temperature the procedure was repeated until the desired amount of sulfur was achieved.

### Electrode processing, cell assembling and electrochemical testing

A mixture of the carbon/sulfur composite powder (83 wt %), Super C65 (Timcal, 6 wt %), Printex XE2 (Orion, 6 wt %) and poly(vinyl alcohol) Selvol 425 (Sekisui, 5 wt %) in water, isopropanol and 1-methoxy-2-propanol (65:30:5 weight ratio) was prepared to form a homogeneous slurry. The slurry coating and drying procedure are described elsewhere [47–48]. 50–60 µm thick electrodes with a sulfur loading of approx. 2.0 mg·cm^−2^ were used for testing. Sulfur cathode, polyethylene membrane (Toray Tonen, 15 mm) and lithium foil (Chemetall Foote Corp., 50 µm) were assembled in coin-type cells inside an argon-filled glovebox from MBraun. The electrolyte used was a solution of lithium bis(trifluoromethanesulfonyl)imide (Aldrich, 99.95%, 8 wt %), lithium nitrate (Merck, 99.995%, 4 wt %), 1,2-dimethoxyethane (Alfa Aesar, >99%, 44 wt %), and 1,3-dioxolane (Acros, 99.8%, 44 wt %). The volume of electrolyte used in the cell was 10 μL/mg_sulfur_. Galvanostatic measurements were performed at 25 °C in the potential range of 2.5–1.7 V versus Li/Li^+^ using a MACCOR Series 4000 (Tulsa, Oklahoma) multichannel battery cycler. A constant voltage step was applied at the end of charging until a current drop of 90% was achieved. Capacity values in the manuscript were calculated on the basis of the sulfur mass. After the formation cycle at a C/50 rate (with 1C = 1672 mA/g_sulfur_) was completed, the cells were charged and discharged at C/5.

### Characterization methods

Dynamic light scattering was measured with a Malvern Nano ZS using a HeNe gas laser with a wavelength of 633 nm.

X-ray diffraction patterns were recorded with a PANalytical X'Pert Pro MPD using Cu Kα radiation (λ = 1.5406 Å, 45 kV, 40 mA).

Thermogravimetric measurements of the samples were carried out on a Netzsch STA 409 at a heating rate of 5 °C/min in air.

Nitrogen physisorption measurements were performed on a Quantachrome Quadrasorb-SI-MP instrument at 77.4 K. Pore size distributions were calculated from the adsorption branch by a non-local density functional theory (NLDFT) model assuming cylindrical pores for silica samples and by a quenched solid density functional theory model assuming slit pores for pores smaller than 2 nm and cylindrical pores for pores larger than 2 nm for carbon samples. The specific surface area was calculated from the adsorption branch in a relative pressure interval from 0.07 to 0.30 by the BET method. Carbon dioxide physisorption was conducted on a Quantachrome Autosorb-iQ-MP instrument at 273.15 K. The pore size distribution was calculated by an NLDFT model assuming slit pores with a moving point average of three. Degassing prior to the physisorption measurements was carried out in vacuum at 120 °C for 20 h for carbon samples and at room temperature for 20 h for carbon/sulfur composites.

Scanning electron microscopy images were taken on a Zeiss Leo 1525 microscope operated at 5 kV and 20 kV for EDX analysis. A carbon layer of approximately 20 nm was deposited onto the samples prior to EDX measurements.

Transmission electron microscopy was carried out using a JEOL JEM 2200 FS operated at 200 kV.

Raman spectra were recorded on a Bruker Senterra Raman microscope (λ = 532 nm, *P* = 2 mW).

## Supporting Information

Supporting Information contains characterization of the silica template (SEM, pXRD, nitrogen physisorption), CO_2_ physisorption of HCS as well as thermal analysis, SEM/EDX analysis and nitrogen physisorption of HCS/sulfur composites and derivation of Equation 3 and Equation 4.

File 1 Additional Information.
