# Supplementary material for: Mesoporous hollow carbon spheres for lithium–sulfur batteries: distribution of sulfur and electrochemical performance
Source: Beilstein J Nanotechnol. 2016 Aug 30;7:1229–40. doi: 10.3762/bjnano.7.114 (PMC5082344; doi:10.3762/bjnano.7.114)
Supplement: File 1 — Additional Information. [file Beilstein_J_Nanotechnol-07-1229-s001.pdf]

Supporting Information

for

Mesoporous hollow carbon spheres for lithium–sulfur batteries: distribution of sulfur and electrochemical performance

Anika C. Juhl[1], Artur Schneider[2], Boris Ufer[1], Torsten Brezesinski[2], Jürgen Janek*[2,3] and Michael Fröba*[1]

Address: [1]Institute of Inorganic and Applied Chemistry, University of Hamburg, Martin-Luther-King-Platz 6, 20146 Hamburg, Germany, [2]Battery and Electrochemistry Laboratory, Institute of Nanotechnology, Karlsruhe Institute of Technology, Hermann-von-Helmholtz-Platz 1, 76344 Eggenstein-Leopoldshafen, Germany and [3]Institute of Physical Chemistry, Justus-Liebig-University Giessen, Heinrich-Buff-Ring 17, 35392 Giessen, Germany

Email: Jürgen Janek* - juergen.janek@phys.chemie.uni-giessen.de;

Michael Fröba* - michael.froeba@chemie.uni-hamburg.de

* Corresponding author

Additional Information

Characterization of silica spheres with a solid core and a mesoporous shell

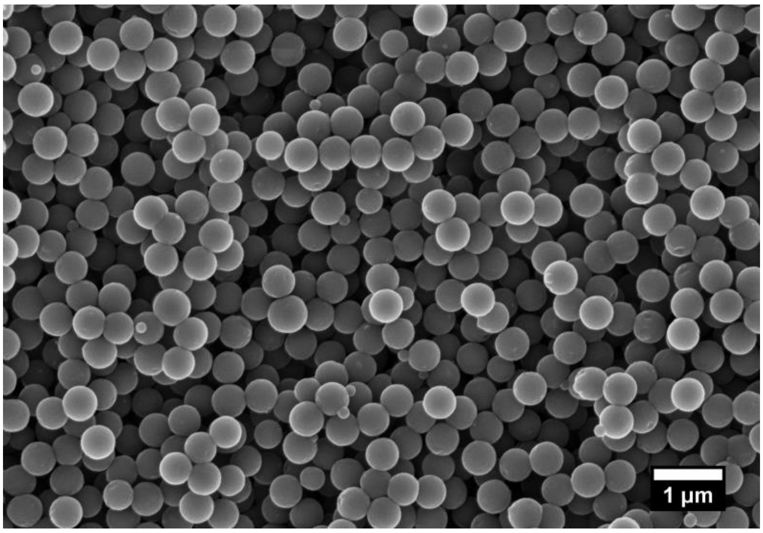

Figure S1: SEM image of monodisperse silica spheres with a solid core and a mesoporous shell. The diameter of the solid core was determined to be 380 nm by dynamic light scattering, while the diameter of the core–shell particles was about 515 nm. From SEM images a diameter of about 490 nm was determined for the core–shell spheres.

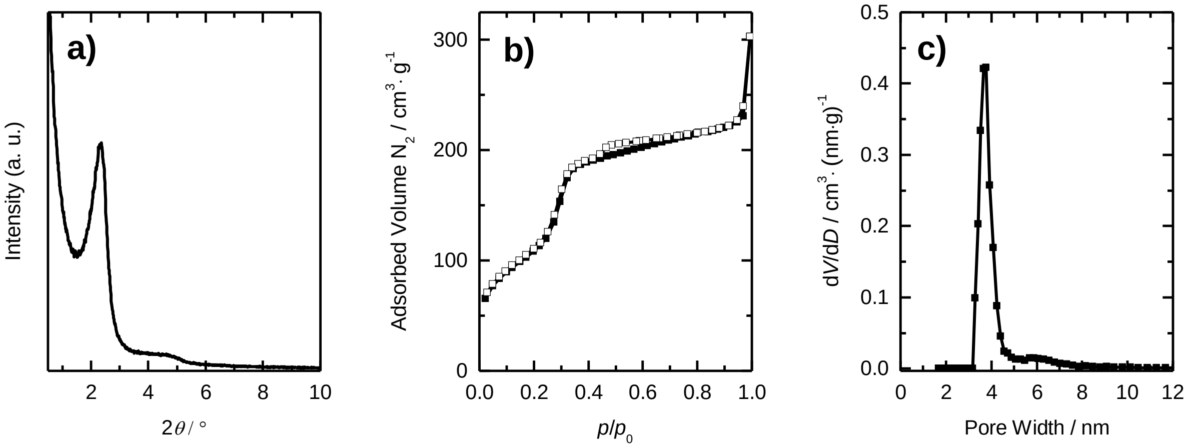

Figure S2: a) Powder X-ray diffraction (XRD) pattern of silica spheres with a solid core and a mesoporous shell, b) nitrogen physisorption isotherm (measured at 77.4 K) and c) pore size distribution calculated by non-local density functional theory (NLDFT).

The powder XRD pattern shows two diffraction peaks at $2\theta = 2.34°$ and $2\theta = 4.67°$. These can be assigned to the (100) and (200) diffraction peaks of a wormlike pore structure. In literature this pattern is assigned to a hexagonal pore structure in which the (110) reflection is scarcely pronounced because of the curved shell [1,2]. From the nitrogen physisorption isotherm a surface of 404 m^2/g can be determined by the Brunauer–Emmett–Teller method. The pore size distribution is narrow and centered at 3.78 nm.

Characterization of hollow carbon spheres

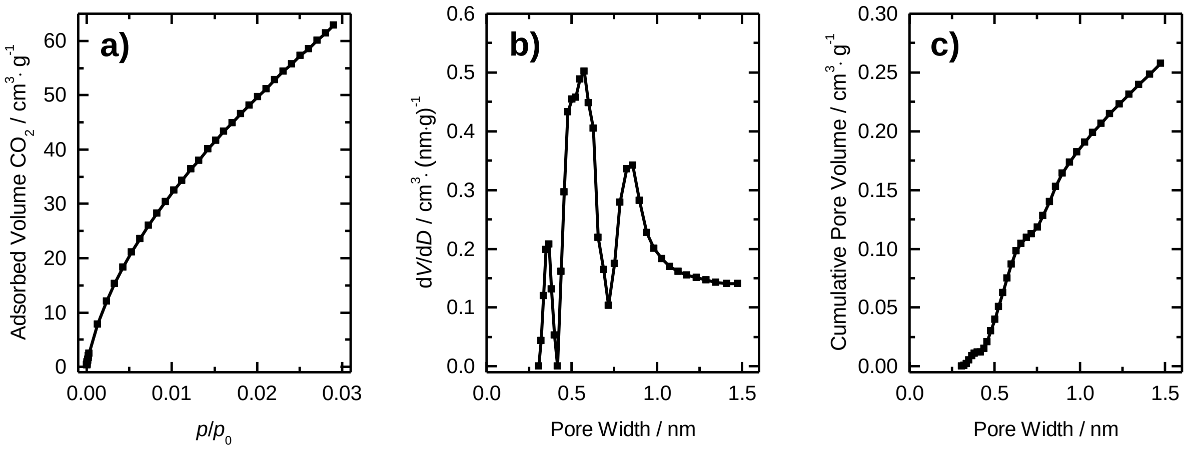

Figure S3: a) Carbon dioxide physisorption isotherm (measured at 273.15 K) of hollow carbon spheres (HCS), b) pore size distribution and c) cumulative pore volume. Pore size distribution and cumulative pore volume were obtained from the isotherm by NLDFT analysis.

From the pore size distribution and plot of cumulative pore volume it can be seen that HCS contain a considerable amount of pores smaller than 1.5 nm. The cumulative pore volume of these small pores is as high as 0.26 cm^3/g.

Calculation of the total possible sulfur loading in HCS

a)

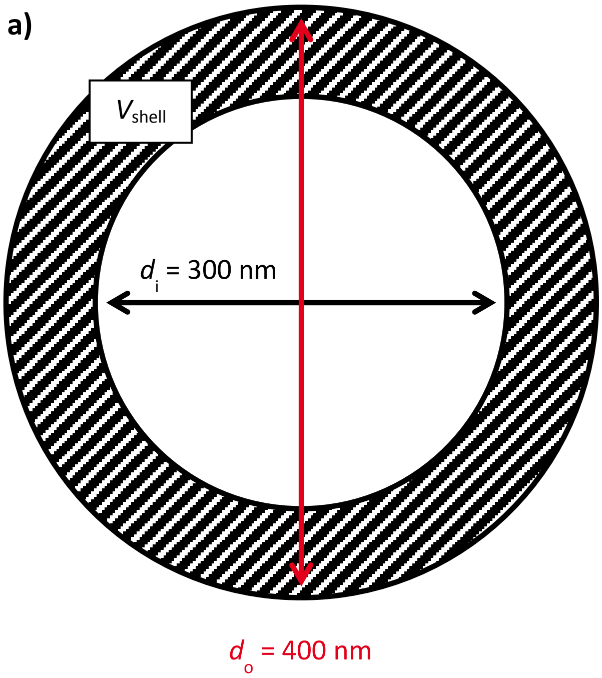

b)

Equations derived from the formula for the volume of a sphere:

$$V_o = \frac{4}{3}\pi r_o^3 \quad (1)$$

$$V_i = \frac{4}{3}\pi r_i^3 \quad (2)$$

$$V_{shell} = \frac{4}{3}\pi\left(r_o^3 - r_i^3\right) \quad (3)$$

Figure S4: a) Schematic presentation of a hollow carbon sphere with an inner diameter d_i, an outer diameter d_o and a volume of the shell V_{shell}. b) Formulas for the volumes of the outer and inner sphere V_o and V_i and the volume of a single shell V_{shell} calculated from that.

The basic equation for calculating the sulfur loading is Equation 4.

$$w_{sulfur} = \frac{m_{sulfur}}{m_{sulfur} + m_{HCS}} \quad (4)$$

Expressing the mass of sulfur by its volume V_{sulfur} and density ρ_{sulfur} (Equation 5) leads to Equation 6.

$$m_{sulfur} = V_{sulfur} \cdot \rho_{sulfur} \quad (5)$$

$$w_{sulfur} = \frac{V_{sulfur} \cdot \rho_{sulfur}}{V_{sulfur} \cdot \rho_{sulfur} + m_{HCS}} \quad (6)$$

The volume of sulfur in the case of complete pore filling can be described by the sum of the volume of all cavities $V_{cavities}$ in 1 g of HCS and the pore volume of the shells V_{pores} in 1 g of HCS.

$$V_{sulfur} = V_{cavities} \cdot V_{pores} \quad (7)$$

In Equation 7 V_{pores} is known from the calculation of the pore volume in the shell (see regular article) while V_{cavities} can be determined by multiplication of the volume of a single cavity V_{i} with the number of spheres N_{spheres} in 1 g of HCS.

$$V_{\text{cavities}} = N_{\text{spheres}} \cdot V_{\text{i}} \tag{8}$$

From here on the geometry of a sphere can be taken into account. The equations that will be used are summarized in Figure S4. The volume of the inner sphere V_{i} of a single hollow sphere is given by Equation 3. The number of spheres can be derived by dividing the volume of all shells V_{shells} by the volume of a single shell V_{shell} (Equation 3 in Figure S4).

$$N_{\text{spheres}} = \frac{V_{\text{shells}}}{V_{\text{shell}}} \tag{9}$$

The volume of all shells is amounted to by the volume of the pure carbon V_{carbon} and the volume of the pores in the shell V_{pores}.

$$V_{\text{shells}} = V_{\text{carbon}} + V_{pores} \tag{10}$$

The volume of the carbon can be calculated from the mass of HCS m_{HCS} and the density of carbon ρ_{carbon}.

$$V_{\text{carbon}} = \frac{m_{\text{HCS}}}{\rho_{\text{carbon}}} \tag{11}$$

By inserting Equations 7–11 in Equation 6, Equation 12 is derived to calculate the total possible sulfur loading.

$$w_{\text{sulfur}} = \frac{\left[\left(\frac{m_{HCS}}{\rho_{carbon}} + V_{pores}\right)\frac{r_i^3}{r_o^3 - r_i^3} + V_{\text{pores}}\right]\cdot \rho_{\text{sulfur}}}{\left[\left(\frac{m_{HCS}}{\rho_{carbon}} + V_{pores}\right)\frac{r_i^3}{r_o^3 - r_i^3} + V_{\text{pores}}\right]\cdot \rho_{\text{sulfur}} + m_{\text{HCS}}} \tag{12}$$

Characterization of HCS/sulfur composites

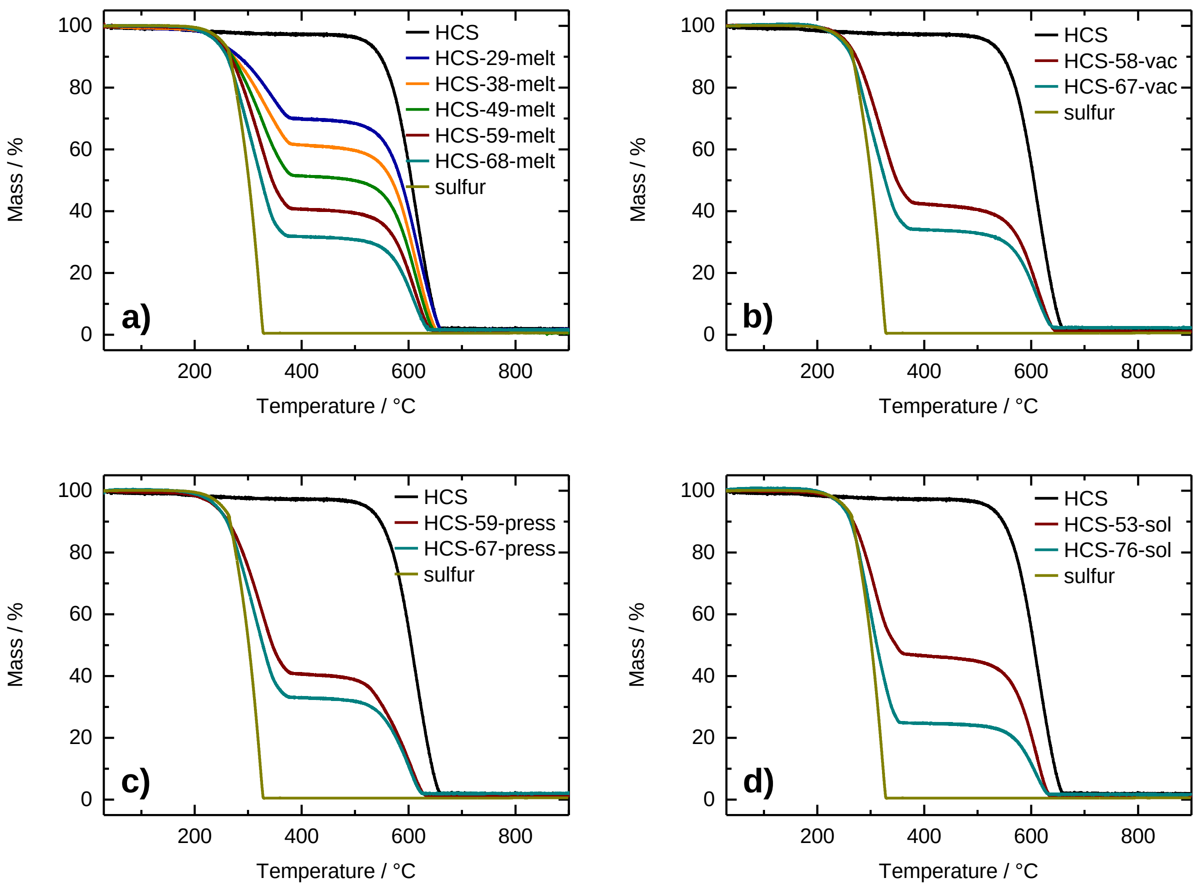

Figure S5: Thermogravimetric measurements (in air, heating rate: 5 °C/min) of HCS/sulfur composites obtained by melt impregnation a) at ambient pressure, b) in vacuum, c) under increased pressure and d) by impregnation from a solution of sulfur in carbon disulfide. From the two distinct steps of sulfur and carbon decomposition the mass fractions of sulfur and carbon in the composites can be determined.

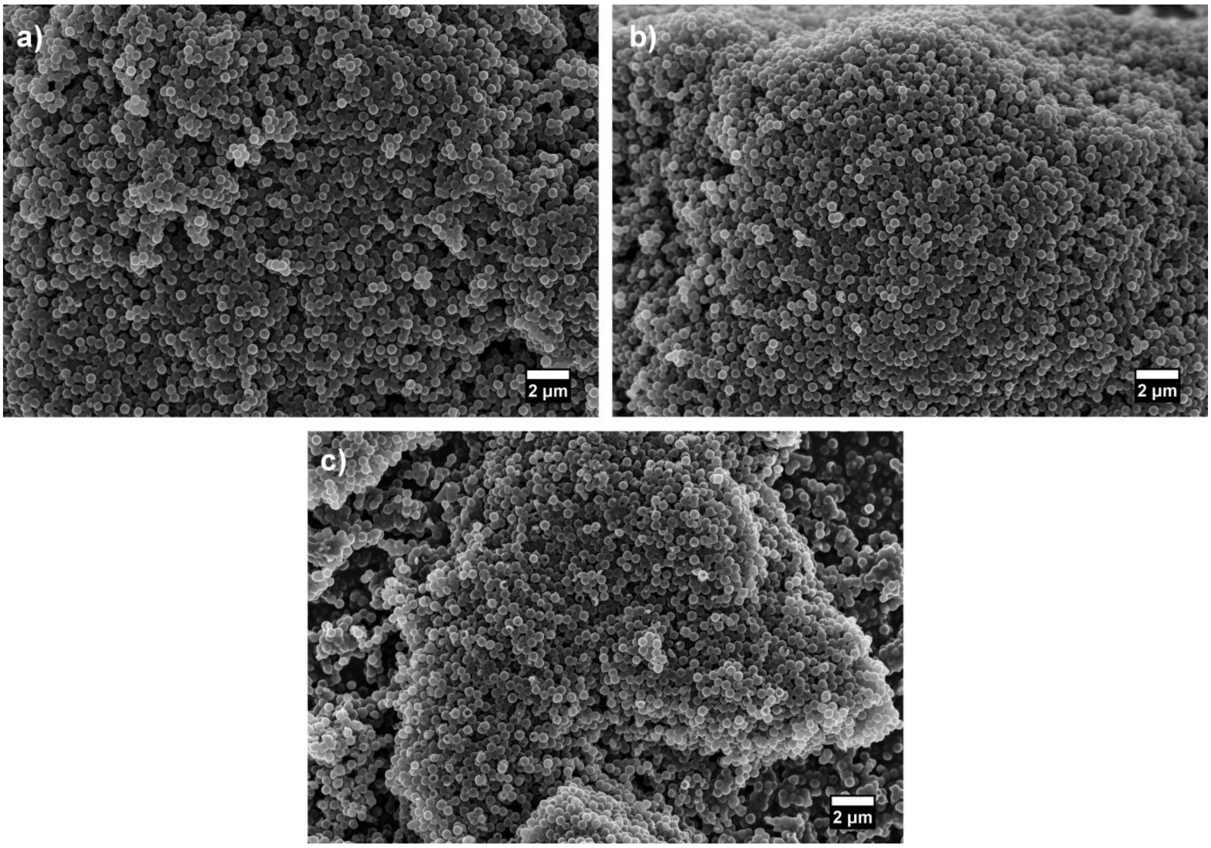

Figure S6: SEM images of HCS/sulfur composites obtained by melt impregnation a) in vacuum (58 wt % sulfur, HCS-58-vac), b) under increased pressure (59 wt % sulfur, HCS-59-press) and c) by impregnation from a solution of sulfur in carbon disulfide (56 wt % sulfur, HCS-56-sol). In none of the images additional sulfur can be found outside the carbon spheres.

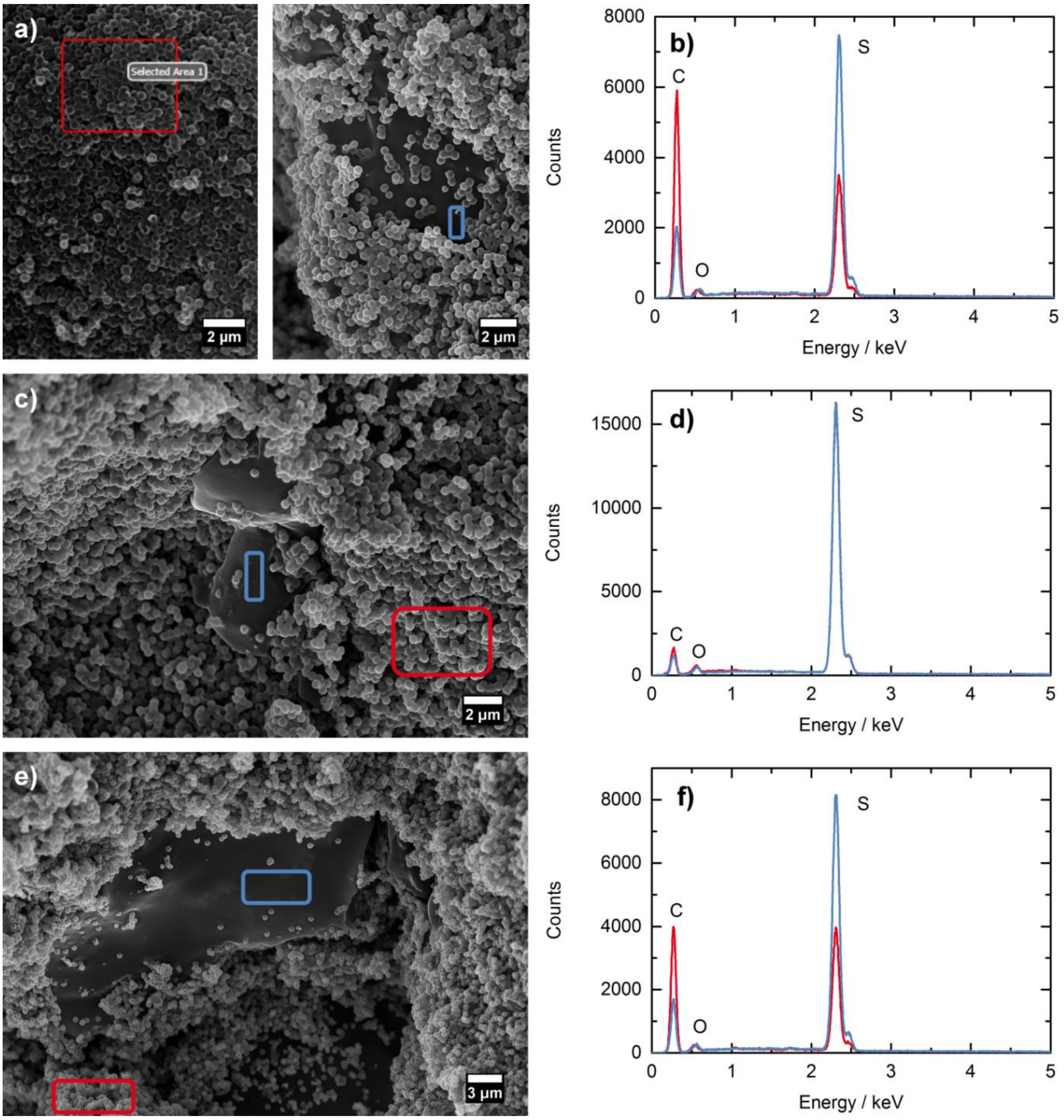

Figure S7: SEM images and EDX measurements of HCS/sulfur composites obtained by melt impregnation a, b) at ambient pressure (68 wt % sulfur, HCS-68-melt), c, d) under increased pressure (67 wt % sulfur, HCS-67-press) and e, f) by impregnation from a solution of sulfur in carbon disulfide (76 wt % sulfur, HCS-76-sol). The measured areas are marked in the images by corresponding colors. If the amounts of carbon and sulfur are compared it becomes obvious that the darker areas in the SEM images contain more sulfur than the HCS. Thus, it can be assumed that they consist of molten and recrystallized sulfur.

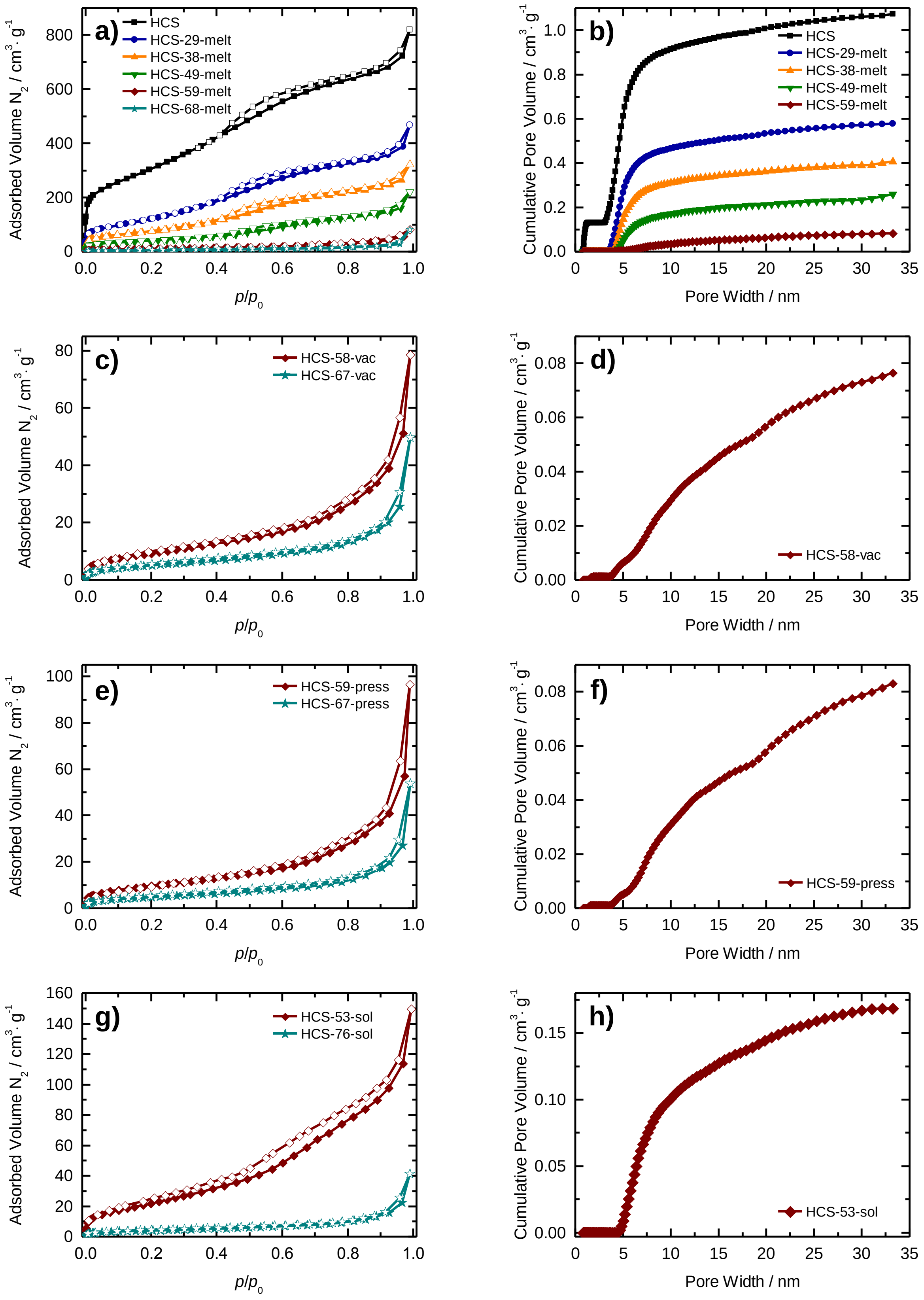

Figure S8: Nitrogen physisorption isotherms (measured at 77.4 K) of the HCS/sulfur composites (a, c, e, g) and cumulative pore volumes of the composites containing up to 60 wt % sulfur calculated by a quenched solid density functional theory model (b, d, f, h). The pore volumes of composites containing more sulfur were not calculated because the pores in the shell are supposed to be completely filled.

Calculation of the theoretical pore volume of HCS/sulfur composites

The theoretical pore volume in the shell of a HCS/sulfur composite $V_{\text{pores,HCS-S}}$ can be calculated by subtracting the volume of sulfur V_{sulfur} from the pore volume of the shell of the hollow spheres V_{pores}.

$$V_{\text{pores,HCS-S}} = V_{\text{pores}} - V_{\text{sulfur}} \tag{13}$$

The volume of sulfur can easily be derived from the mass and density of sulfur (Equation 14), while the pore volume of the shell is obtained by multiplying the specific pore volume $V'_{\text{pores,HCS}}$ by the mass of HCS m_{HCS} (Equation 15).

$$V_{\text{sulfur}} = \frac{m_{\text{sulfur}}}{\rho_{\text{sulfur}}} \tag{14}$$

$$V_{\text{pores}} = V'_{\text{pores,HCS}} \cdot m_{\text{HCS}} \tag{15}$$

When the Equations 14 and 15 are inserted into Equation 13, Equation 16 is obtained.

$$V_{\text{pores,HCS-S}} = V'_{\text{pores,HCS}} \cdot m_{\text{HCS}} - \frac{m_{\text{sulfur}}}{\rho_{\text{sulfur}}} \tag{16}$$

For the specific pore volume of the composite $V'_{\text{pores,HCS-S}}$ Equation 16 has to be divided by the mass of the composite $m_{\text{HCS}} + m_{\text{sulfur}}$.

$$V'_{\text{pores,HCS-S}} = \frac{V'_{\text{pores,HCS}} \cdot m_{\text{HCS}} - \frac{m_{\text{sulfur}}}{\rho_{\text{sulfur}}}}{m_{\text{HCS}} + m_{\text{sulfur}}} \tag{17}$$

References

1. Yoon, S. B.; Kim, J.-Y.; Kim, J. H.; Park, Y. J.; Yoon, K. R.; Park, S.-K.; Yu, J.-S. *J. Mater. Chem.* **2007**, *17*, 1758–1761.
2. Kim, J. H.; Yoon, S. B.; Kim, J.-Y.; Chae, Y. B.; Yu, J.-S. *Colloids Surfaces A Physicochem. Eng. Asp.* **2008**, *313-314*, 77–81.
